# Open-Ended Learning: A Conceptual Framework Based on Representational Redescription

**DOI:** 10.3389/fnbot.2018.00059

**Published:** 2018-09-25

**Authors:** Stephane Doncieux, David Filliat, Natalia Díaz-Rodríguez, Timothy Hospedales, Richard Duro, Alexandre Coninx, Diederik M. Roijers, Benoît Girard, Nicolas Perrin, Olivier Sigaud

**Affiliations:** ^1^Sorbonne Université, CNRS, ISIR, Paris, France; ^2^U2IS, INRIA Flowers, ENSTA ParisTech, Palaiseau, France; ^3^Institute of Perception, Action and Behaviour, University of Edinburgh, Edinburgh, United Kingdom; ^4^GII, Universidade da Coruña, A Coruña, Spain; ^5^Department of Computer Science, Vrije Universiteit Amsterdam, Amsterdam, Netherlands

**Keywords:** developmental robotics, reinforcement learning, state representation learning, representational redescription, actions and goals, skills

## Abstract

Reinforcement learning (RL) aims at building a policy that maximizes a task-related reward within a given domain. When the domain is known, i.e., when its states, actions and reward are defined, Markov Decision Processes (MDPs) provide a convenient theoretical framework to formalize RL. But in an open-ended learning process, an agent or robot must solve an unbounded sequence of tasks that are not known in advance and the corresponding MDPs cannot be built at design time. This defines the main challenges of open-ended learning: how can the agent learn how to behave appropriately when the adequate states, actions and rewards representations are not given? In this paper, we propose a conceptual framework to address this question. We assume an agent endowed with low-level perception and action capabilities. This agent receives an external reward when it faces a task. It must discover the state and action representations that will let it cast the tasks as MDPs in order to solve them by RL. The relevance of the action or state representation is critical for the agent to learn efficiently. Considering that the agent starts with a low level, task-agnostic state and action spaces based on its low-level perception and action capabilities, we describe open-ended learning as the challenge of building the adequate representation of states and actions, i.e., of redescribing available representations. We suggest an iterative approach to this problem based on several successive Representational Redescription processes, and highlight the corresponding challenges in which intrinsic motivations play a key role.

## 1. Introduction

Robots need world representations in terms of objects, actions, plans, etc. Currently such representations are carefully designed and adapted to the robot's task (Kober et al., 2013). But a general purpose robot capable of solving an unbounded number of tasks cannot rely on representations hardwired at design time, because each may require a different representation. To achieve the vision of a robot that can solve an open-ended series of tasks in an increasingly efficient way, we consider an alternative paradigm: that the robot should discover the appropriate representations required to autonomously learn each task.

Representational redescription is the ability to discover new representations based on existing ones. It is a key ability of human intelligence (Karmiloff-Smith, 1995) that remains a challenge in robotics (Oudeyer, 2015). In this paper, we propose a unifying conceptual framework for addressing it. We assume an agent endowed with low-level perception and action capabilities which receives external rewards when it faces a task. We also assume the agent is endowed with reinforcement learning (RL) capabilities efficient enough to let it learn to solve a task when cast as a Markov Decision Process (MDP). From these assumptions, the main challenge in our framework is determining how an agent can discover the state and action representations that let it cast tasks as MDPs, before solving them by RL (Zimmer and Doncieux, 2018).

**Figure 1:**
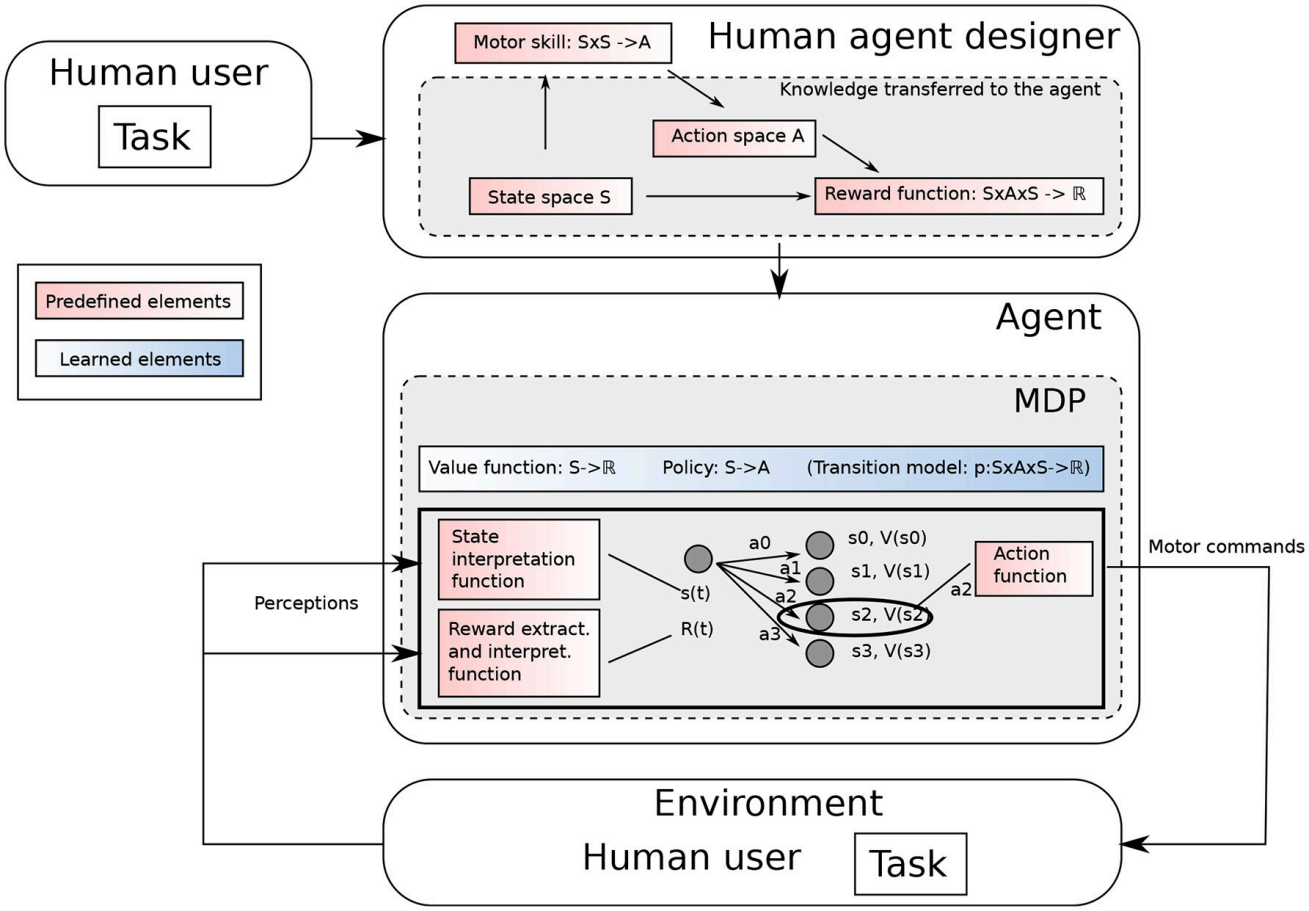
A typical MDP. The agent designer having a task in mind designs the MDP accordingly.

In MDPs, states and actions are primitive components considered given, and they are generally defined by the human designer having a particular task and domain in mind (see Figure 1). To make a step toward open-ended learning, we propose a conceptual framework for representational redescription processes based on a formal definition of states and actions. Then we highlight the challenges it raises, notably in terms of intrinsic motivations.

## 2. The representational redescription approach

**Figure 2:**
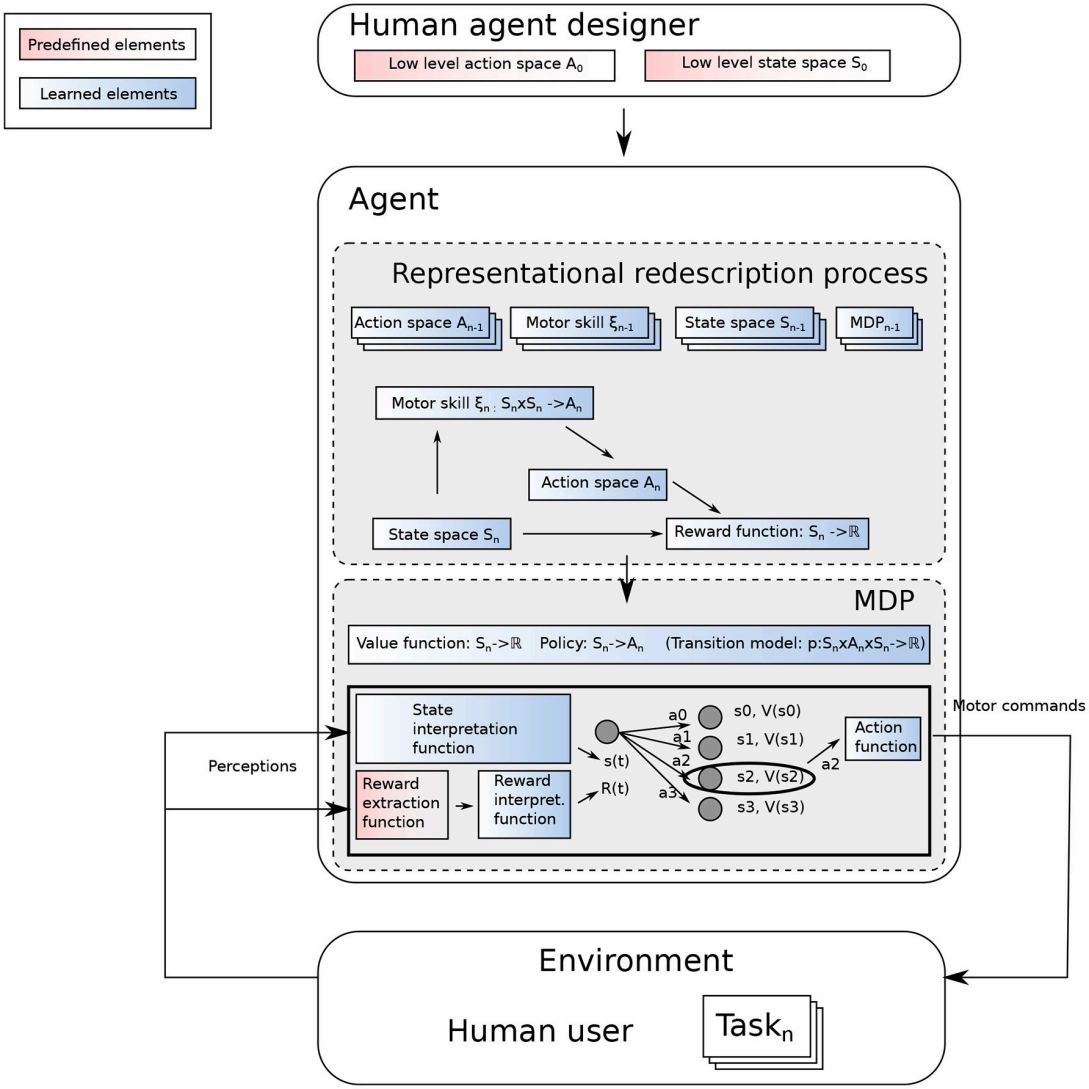
Overview of an open-ended learning process. The agent designer does not know the different tasks the agent will be facing, but designs the agent to let it build the MDP to interpret a reward in its environment and find out how to maximize it.

Our Representational Redescription approach is depicted in Figure 2. We consider an agent endowed with low-level perception and action capabilities, and which faces an open-ended sequence of tasks. The agent receives some external rewards from these tasks. The problem for this agent is to determine how to use this reward to learn the corresponding task. In an MDP, an RL algorithm explores the possible outcomes of an action when executed in a particular state. As pointed out by Kober et al. (2013), there is a need to appropriately define the state and action spaces for an efficient learning process. To do so, the possible alternatives are either to rely on a single generic state and action space or to build them on-the-fly when required. In this work, we do the latter and make the following assumptions:

ASSUMPTION 1. *A* single *state and action space cannot efficiently represent all the decision processes required to solve the tasks an open-ended learning system will be confronted to. To solve the task *k* defined through a reward value *r*_*k*_(*t*), the agent needs to build an MDP M_k_*.

ASSUMPTION 2. *An open-ended learning process needs to build these MDPs on-the-fly*.

ASSUMPTION 3. *The agent is endowed with some RL algorithms to allow it to learn to solve the task, once the underlying MDP has been fully defined*.

## 3. Conceptual framework and basic definitions

### 3.1. Markov decision processes

Decisions in robotics can be modeled with MDPs using < *S*_*k*_, *A*_*k*_, *p*_*k*_, *R*_*k*_>, where *k* is a task identifier^1^, *S*_*k*_ is the state space, *A*_*k*_ is the action space, *p*_*k*_:*S*_*k*_×*A*_*k*_×*S*_*k*_ → ℝ is a transition function, where *p*_*k*_(*s*_*t*_, *a*_*t*_, *s*_*t*+1_) gives the probability to reach *s*_*t*+1_ from *s*_*t*_ after having applied action *a*_*t*_ and *R*_*k*_:*S*_*k*_ → ℝ is the reward function. A policy π_*k*_:*S*_*k*_→*A*_*k*_ is a process that determines which action to apply in any state.

In the proposed framework, the observed reward *r*_*k*_(*t*) is distinguished from the reward function of the MDP *R*_*k*_(*t*). The agent may not know to what state the observed reward *r*_*k*_(*t*) can be associated. It is actually part of the proposed open-ended learning framework to *interpret* observed rewards and associate them to states in order to build the reward function *R*_*k*_(*t*) required to learn how to maximize them.

The notations used here have been intentionally kept as simple as possible. This framework can be easily extended to more complex cases, including semi-MDPs, stochastic policies or other definitions of the reward function.

### 3.2. States

DEFINITION 1. *A* state *is a description of a robot context that respects the constraints of its decision process*.

Following (Lesort et al., 2018), a good state representation should be (1) Markovian (i.e., the current state summarizes all the necessary information to choose an action), (2) able to represent the robot context well enough for policy improvement, (3) able to generalize the learned value-function to unseen states with similar features, and (4), low dimensional for efficient estimation (Böhmer et al., 2015). State representation learning approaches learn low dimensional representations without direct supervision, i.e., exploiting sequences of observations, actions, rewards and generic learning objectives (Lesort et al., 2018).

To bootstrap the open-ended learning process, we define *S*_0_ as the state space containing the set of possible sensorimotor values. This first state space may not be low dimensional, Markovian, or structured enough for efficient exploration, thus motivating the search for better adapted state spaces.

### 3.3. Reward functions and goals

A reward function may contain different kinds of information: an indication of success in fulfilling a Human user defined task, or in reaching an autonomously defined action goal (see next section). It may also contain guidance to help reach the goal (reward shaping).

Besides reward functions defined in ℝ, the proposed framework requires, for the description of actions, the definition of boolean reward functions that will be called *goal functions*:

DEFINITION 2. *A* goal function, *denoted* $\hat{R}$, *does not contain any shaping term and tells whether the goal associated to this reward function is achieved or not*.

A goal function is a specific reward function aimed at defining the notion of success or failure required for action definition. The task to solve does not need to be described with such a boolean function.

DEFINITION 3. Goal states *are states s for which* $\hat{R}\left(s\right)=True$.

### 3.4. Actions

In the proposed framework, actions are not systematically predefined, but can be built on-the-fly. The design of the corresponding algorithms requires to define what an action actually is. The proposed definition relies on the notion of goal function to add a purpose to a policy. Actions are framed within different abstraction levels depending on the granularity of the policy, as in the options framework (Sutton et al., 1999). Actions are one of the main components of an MDP. An MDP *M*_*k*_ needs an action space *A*_*k*_. *A*_*k*_ is an action space defined at an abstraction level *k*. It relies on policies of level *k*−1, defined in an MDP *k*−1. They can be used to build policies at the level *k* that can, in turn, be used to build new actions for another MDP at the level *k*+1.

DEFINITION 4. *Actions a∈A_k_ are the primitives of MDP M_k_*. *An* action *a is a policy π relying on actions available at a lower level and built to reach a goal state associated to a goal function $\hat{R}$. The action succeeds if the trajectory of the robot controlled by this policy converges to a goal state of $\hat{R}$; otherwise, it fails. An action is then fully defined by the triplet: $\left\{\pi ,\hat{R},t_{max}\right\}$ where t_max_ is the maximum amount of time after which the action is considered failed if no goal state is reached*.

If the level on top of which an MDP *M*_*k*_ is built is, itself, an MDP, actions *a*∈*A*_*k*_ can be considered as macro-actions or options.

The goal state of an action is frequently defined relative to a particular initial state *s*_*init*_, where *s*_*init*_ is the state of the agent when the action is triggered, e.g., “Turning 90*deg*” or “moving forward 1*m*.”

The definition of an action is hierarchically recurrent: an action *a*_*k*_ relies on a policy π that also relies on a set of lower level actions {*a*_*l*_, *l*<*k*}. To stop the recurrence, a specific set of actions *A*_0_ is defined, that corresponds to the lowest level accessible by the robot, i.e., motor commands. They are also actions, as motor commands always have a goal (reaching a particular velocity or position, for instance) that a low-level control process aims at reaching and eventually staying at. As suggested by Harutyunyan (2018, Chapter 5), we assert that it may not be necessary, or even desirable, to have the same time-scale and discounting for lower and higher level actions.

### 3.5. Representational redescription

In the proposed framework, open-ended learning needs to build MDPs on-the-fly, including the state and action spaces. Considering that the process starts from initial state and action spaces (*S*_0_, *A*_0_), this particular feature is captured by the concept of *representational redescription*.

DEFINITION 5. *A* representational redescription process *is a process that builds the state and action spaces enabling the definition of an MDP able to (1) solve a given task defined by observed reward values (2) in a particular domain and (3) with a particular decision or learning process. To this end, it relies on the representations of states and actions that have been previously acquired and can thus be described as a process transforming existing representations into new ones that are more fitted to the context*.

### 3.6. Motor skills: controlling transitions between states

In an MDP, the set of provided actions is built to allow the robot to move in the state space. If a state space is built on-the-fly, the agent should be able to control it and move from one state to another. With the proposed definitions, the open-ended learning process needs to build actions to reach each part of the state space. The notion of motor skill is defined to capture this process.

DEFINITION 6. *A* motor skill *is an action generator:* $\xi_{k}:S^{\left(i\right)}\times S^{\left(g\right)}\to A_{k}$, *where* $S^{\left(i\right)},S^{\left(g\right)}\subset S_{k}^{2}$. *It is an inverse model defined in a particular action space A_k_ to reach a target state from an initial state*.

ξ(*s*_*i*_, *s*_*g*_) is an action that, starting from $s_{i}\in S^{\left(i\right)}$, reaches $s_{g}\in S^{\left(g\right)}$ with the highest possible probability. The state *s*_*g*_ is the goal state of the corresponding action, and the corresponding reward function is intrinsic (see section 3.8).

### 3.7. Open-ended learning

On the basis of the proposed definitions, we can define an open-ended learning process as follows:

DEFINITION 7. *An* open-ended learning process *builds the MDPs required to solve the tasks that the agent faces. Task k is defined through an observed reward value r_k_(t). Starting from an initial state space S_0_, an initial action space A_0_ and a decision or learning process P, the open-ended learning process aims at building (1) state spaces, (2) action spaces and (3) motor skills to control the state with appropriate actions. State and action spaces need to fulfil the following features*:

1. The state space should help interpret the reward occurences, i.e., learn R_k_ to model the observed r_k_;
2. The action space should allow control of the state space through dedicated motor skills;
3. The state and action spaces should make the agent's state trajectory as predictable as possible;
4. *From the state and action spaces*, P *should be able to converge to a policy maximizing r_k_*.

### 3.8. Intrinsic motivations

Task-based rewards are not enough to drive a representational redescription process. There is a need for other drives that push the agent to explore and create new knowledge. This is the role of intrinsic motivations (Oudeyer and Kaplan, 2009; Baldassarre and Mirolli, 2013). In the context of open-ended learning through representational redescription, we propose the following definition:

DEFINITION 8. *An* intrinsic motivation *is a drive that complements drives associated with external task-based rewards to build appropriate state and actions spaces as well as motor skills*.

Intrinsic motivations play a critical role at different stages of the proposed representational redescription process, for instance:

- To organize learning and select in what order to learn skills and build state spaces;
- To acquire relevant data for state representation learning (before building an appropriate MDP);
- To build the skills required to control the state space (focusing learning on areas that are within reach and ignoring the rest).

## 4. Challenges

This section recasts the challenges of open-ended learning with the proposed conceptual framework.

CHALLENGE 1. *Interpreting observed reward: Building an appropriate state space to interpret an externally provided reward, i.e., build a state space *S*_*k*_ that allows easy modeling of the observed reward value *r*_*k*_*.

CHALLENGE 2. *Skill acquisition: Controlling the displacements in an acquired state space *S*_*k*_ by building the appropriate action space *A*_*k*_ and skill $\xi_{k}:S^{\left(i\right)}\times S^{\left(g\right)}\to A_{k}$, where $S^{\left(i\right)},S^{\left(g\right)}\subset S_{k}^{2}$, to give the agent the ability to move from one state to another as accurately as possible*.

To address Challenge 1, state representations can be learned from known actions (Jonschkowski and Brock, 2015) and, likewise, to address Challenge 2, actions can be learned when the state space is known (Rolf et al., 2010; Forestier et al., 2017).

CHALLENGE 3. *Simultaneously learning state space, action space, and policy: The state and action spaces are interdependent with each other and with the policy. For open-ended learning, all need to be learned jointly to solve a task, and doing so tractably is a challenge*.

CHALLENGE 4. *Dealing with sparse reward: available state and action spaces may not allow to easily obtain reward *r*_*k*_(*t*) associated to Task *k*. This is particularly true at the beginning of the process, when starting from (*S*_0_, *A*_0_): this is the bootstrap problem. The challenge is to design an exploration process that converges toward reward observations in a limited time*.

A possibility to address the bootstrap problem is to rely on a motor babbling approach (Baranes and Oudeyer, 2010; Rolf et al., 2010). Another possibility would be to rely on a direct policy search including an intrinsic motivation toward behavior diversity and followed by a process to extract adapted representations from it (Zimmer and Doncieux, 2018).

The next challenges are related to the unsupervised acquisition of a hierarchy of adapted representations.

CHALLENGE 5. *Detecting task change: In the case where tasks are not explicitly indicated to the robot, detecting task change from *k* to *k*+1 on the basis of observed rewards r*.

The efficiency of a learning system is influenced by the order of the tasks it is facing (Bengio et al., 2009).

CHALLENGE 6. *Ordering knowledge acquisition and task resolution: An open-ended learning system needs to be able to select what to focus on and when. Does it keep learning representations for task *k* (even if *r*_*k*_ has momentarily disappeared), or does it focus on a new task *k*+1 ?*

CHALLENGE 7. *Identifying the available knowledge relevant to build the new representations *MDP*_*k*_: as the set of available MDPs grows, it becomes a challenge to figure out what knowledge can help to build a new and adapted representation, i.e., {*MD*_*P*_*l*_, π_*l*_}*l* ≤ *k*_ = { < _*S*_*l*_, *A*_*l*_, *p*_*l*_, *R*_*l*_>, π_*l*_}*l* ≤ *k*_*.

CHALLENGE 8. *Using transfer learning for speeding up state and action spaces learning along time: as the number of tasks and domains the agent can deal with grows, it becomes interesting when facing a task *k*+1 to reuse the knowledge available to avoid learning *MDP*_*k*+1_ and π_*k*+1_ from scratch*.

## 5. Discussion

In contrast to many works in multitask learning (Zhao et al., 2017; Riedmiller et al., 2018), we assume here that each task should be solved with its own state and action representation, and learning these representations is a central challenge. We adopt a hierarchical perspective based on representational redescription which differs from the hierarchical RL perspective (Barto and Mahadevan, 2003) from the fact that we do not assume that the lowest level is necessarily described as an MDP and we assume that each intermediate level may come with its own representation.

The proposed framework is related to end-to-end approaches to reinforcement learning (Lillicrap et al., 2015; Levine et al., 2016), but instead of black box approaches, it emphasizes knowledge reuse through the explicit extraction of relevant representations.

Open-ended learning is expected to occur in a lifelong learning scenario in which the agent will be confronted with multiple challenges to build the knowledge required to solve the tasks it will face. It will not be systematically engaged in a task resolution problem and will thus have to perform choices that cannot be guided by a reward. Intrinsic motivations are thus a critical component of the proposed open-ended learning system. They will fill in multiple needs of such a system: (1) a drive for action and state space acquisition (Péré et al., 2018), (2) a selection of what to focus on (Oudeyer et al., 2007) and (3) a bootstrap of the process in the case of sparse reward (Mouret and Doncieux, 2012).

## Author contributions

This article is the result of a joint work within the DREAM project. Each author has participated to the discussions that have lead to the proposed formalism. SD has coordinated the discussions and the writing.

### Conflict of interest statement

The authors declare that the research was conducted in the absence of any commercial or financial relationships that could be construed as a potential conflict of interest.

## Footnotes

^1^A single state and action space can be used for several tasks and a single task could be associated to multiple representations, but we use this notation to highlight the dependency between tasks and MDPs that is central to this framework.
